# The Incidence and Characteristics of Perinatal Stroke in Beijing: A Multicenter Study

**DOI:** 10.3389/fpubh.2022.783153

**Published:** 2022-03-24

**Authors:** Qianqian Xia, Zhao Yang, Yao Xie, Ying Zhu, Zixin Yang, Mingyan Hei, Yingxue Ding, Weijing Kong, Limin Kang, Shengnan Yang, Yabo Mei, Zhichun Feng, Li Zhang, Yanzhe Lei, Ping Wang, Jingyu Dong, Li Yang, Jun Ju, Hesheng Chang, Shina Zhan, Jinqian Yu, Peng Zhang, Ran Wang, Hui Guo, Xinli Liu, Huaqing Tan, Yakun Liu, Zhenzong Zhang, Lixia Zhen, Jinting Yan, Zhan Liu, Chunxia Yang, Qingqing Wang, Jinfeng Wang, Lifang Sun, Huihui Zeng, Jing Li, Wenwen Qu, Xuemei Wang, Guiying Liu, Xi Yang, Xinxin Liu, Xuefeng Zhang, Xiaojing Xu, Yanan Gu, Hui Long, Li Zhang, Lili Liu, Zezhong Tang, Xinlin Hou

**Affiliations:** ^1^Pediatric Department, Peking University First Hospital, Beijing, China; ^2^Beijing Children's Hospital, Capital Medical University, National Center for Children's Health, Beijing, China; ^3^Beijing Friendship Hospital, Capital Medical University, Beijing, China; ^4^Department of Neonatology, Children's Hospital, Capital Institute of Pediatrics, Beijing, China; ^5^Neonatal Intensive Care Unit of Faculty of Pediatrics, Chinese People's Liberation Army (PLA) General Hospital, Beijing, China; ^6^Pediatric Department, Haidian Maternal and Child Health Hospital, Beijing, China; ^7^Department of Neonatology, Beijing Obstetrics and Gynecology Hospital, Capital Medical University, Beijing, China; ^8^Pediatric Department, Tongzhou Maternal and Child Health Hospital, Beijing, China; ^9^Pediatric Department, Beijing Chao-Yang Hospital, Capital Medical University, Beijing, China; ^10^Pediatric Department, Shunyi Maternal and Children's Hospital of Beijing Children's Hospital, Beijing, China; ^11^Pediatric Department, Beijing Miyun Maternal and Child Health Hospital, Beijing, China; ^12^Pediatric Department, Fengtai Maternal and Child Health Hospital, Beijing, China; ^13^Pediatric Department, Beijing Daxing Maternal and Child Care Hospital, Beijing, China; ^14^Pediatric Department, The First Hospital of Fangshan, Beijing, China; ^15^Pediatric Department, Beijing Mentougou Hospital, Beijing, China; ^16^Pediatric Department, Changping Women and Children Health Care Hospital, Beijing, China; ^17^Beijing Pinggu Maternal and Child Health Hospital, Beijing, China; ^18^Pediatric Department, Huairou Maternal and Child Health Care Hospital, Beijing, China; ^19^Department of Pediatrics, Peking University Shougang Hospital and Capital Medical University, Beijing, China; ^20^General Respiratory Department of Beijing Jingdu Children's Hospital, Beijing, China; ^21^Department of Pediatrics, Beijing Anzhen Hospital, Capital Medical University, Beijing, China; ^22^Pediatric Department, Fifth Medical Center of Chinese People's Liberation Army (PLA) General Hospital, Beijing, China; ^23^Pediatric Department, The First Hospital of Tsinghua University, Beijing, China; ^24^Department of Pediatrics, Chinese People's Liberation Army (PLA) General Hospital, Beijing, China

**Keywords:** neonatal stroke, incidence, diagnosis, clinical characteristics, prognosis

## Abstract

**Objective:**

To assess the incidence, risk factors, and clinical characteristics of perinatal stroke in Beijing.

**Methods:**

This multicenter prospective study included all the live births from 17 representative maternal delivery hospitals in Beijing from March 1, 2019 to February 29, 2020. Neonates with a stroke were assigned to the study group. Clinical data, including general information, clinical manifestations, and risk factors, were collected. Up until 18 months after birth, neonates were routinely assessed according to the Ages and Stages Questionnaire (ASQ) and/or the Bayley scale. Statistical analysis was done using the chi-squared, *t*-tests, and logistic regression analysis using SPSS version 26.0.

**Outcomes:**

In total, 27 cases were identified and the incidence of perinatal stroke in Beijing was 1/2,660 live births, including 1/5,985 for ischemic stroke and 1/4,788 for hemorrhagic stroke. Seventeen cases (62.96%) of acute symptomatic stroke and convulsions within 72 h (10 cases, 37.04%) were the most common presentations. Ten patients showed no neurological symptoms and were found to have had a stroke through routine cranial ultrasonography after being hospitalized for non-neurological diseases. The risk factors include primiparity, placental or uterine abruption/acute chorioamnionitis, intrauterine distress, asphyxia, and severe infection. In the study group, 11.1% (3/27) of patients had adverse neurodevelopmental outcomes. The patients in the study group had lower scores for the ASQ than those in the control group in the communication, gross, and fine motor dimensions.

**Conclusion:**

The incidence of perinatal stroke in Beijing was consistent with that in other countries. Routine neuroimaging of infants with risk factors may enable identification of asymptomatic strokes in more patients. Patients who have suffered from a stroke may have neurological sequelae; therefore, early detection, treatment, and regular follow-ups are beneficial for improving their recovery outcomes.

## Introduction

Perinatal stroke is a cerebrovascular disease that occurs in newborns from 20 weeks of gestation to 28 days after birth. The incidence of this type of stroke is one in every 1,600–3,000 live births (1–4). This can involve either the arteries and/or veins, being categorized as hemorrhagic stroke and ischemic stroke (1, 5). Specifically, perinatal strokes can be divided into neonatal arterial ischemic strokes, neonatal cerebral venous sinus thrombosis, and neonatal hemorrhagic strokes. Although the incidence of perinatal strokes is low, it seriously increases the likelihood of an added prognosis. Of those children with perinatal strokes, 40% develop epilepsy after the neonatal period (6), 68% of neonates diagnosed with ischemic strokes develop dyskinesia (7), 43% of these children suffer from cognitive dysfunctions (8, 9), and neurological sequelae occurs in 47% of neonates with hemorrhagic strokes (10). Some patients suffer from an acute onset of symptoms, including seizures, changes in tone, irritability, feeding difficulties, and dystonia, which are classified as an acute symptomatic perinatal stroke.

The leading unit of this project, the Peking University First Hospital, has a fully equipped neonatal neurointensive care unit. This unit is one of the main referral centers for neonatal brain injury in Beijing, actively taking part in the referral, treatment, and long-term follow-ups of neonates with these conditions. In the author's experiences, although the incidence of perinatal strokes is lower than that of hypoxic-ischemic encephalopathy, which is the major cause of neurologic disabilities in term neonates, the former is still rather common. When these children are diagnosed early, particularly through imaging screenings for asymptomatic high-risk infants and receive physical therapy later in their childhood, their prognosis could be improved (11).

The main purpose of this study was to assess the incidence, risk factors, and clinical characteristics of perinatal strokes in Beijing. Specifically, it aimed to analyze its high-risk factors, disease onset time, symptoms, and prognosis. The hope was to supply a basis for the diagnosis and treatment of strokes that occur during the perinatal period among children and to improve their future prognosis. The Peking University First Hospital led the study, but it also includes 16 representative maternity hospitals and three neonatal referral hospitals, thus covering the main administrative regions of Beijing.

## Methods

**Figure 1:**
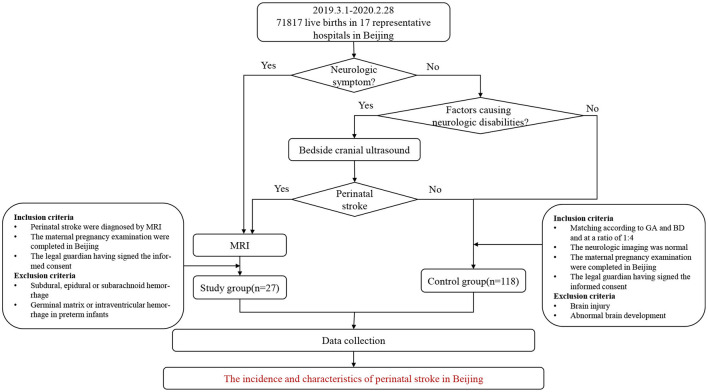
Case ascertainment methods. GA, gestational age; BD, birth date.

This was a multicenter prospective study, with 17 of the recruiting hospitals being maternity hospitals and three neonatal referral hospitals for children with severe illnesses in Beijing. The participating maternal delivery hospitals included secondary and tertiary hospitals that covered the central and suburban areas of the city, all of which are the main delivery hospitals in the administrative region of Beijing. The 17 maternity hospitals then conducted follow-ups on all the newborns, who were alive at birth until the diagnosis of perinatal stroke was either excluded or confirmed. Some infants with neurologic presentations were assessed with MRI and then for further evaluation. Neurological symptoms or signs included, but was not limited to the following manifestations: convulsions, frequent myoclonus, altered consciousness (drowsiness, responsiveness, and irritability), abnormal respiratory rhythm, weakened muscle strength, dystonia, postural abnormalities, feeding difficulties (inappetence and emesis), and the abnormal Neonatal Behavioral Neurological Assessment (NBNA) score. Those who did not display neurological symptoms, but who had risk factors that could lead to neurologic disabilities in an infant, maternal and peripartum characteristics, first underwent a craniocerebral ultrasound. If the ultrasound, with an abnormal echo in parenchyma or sinus, showed symptoms or signs of a suspected perinatal stroke, the neonate would be further assessed with an MRI (Figure 1). If the participating maternity hospital teams believed it too difficult to diagnose, treat, or follow-up with any of the children, they were referred to the Pediatrics Department of Peking University First Hospital or one of the three neonatal referral hospitals for further diagnosis and treatment. The cases of the control group were matched based on the perinatal stroke cases.

Clinical data collection and regular follow-ups were included in the standardized diagnosis and treatment process of the enrolled newborns. The results of the follow-ups and related data were analyzed. This study's enrollment period was from March 1, 2019 to February 29, 2020. This study was approved by the Ethics Committee of Peking University First Hospital and a written informed consent was obtained from the legal guardians of the newborns.

## Patients

### Study Group

All the newborns delivered in the participating units from March 1, 2019 to February 29, 2020 were followed-up by the delivery unit team until 42 days after their birth. If any neurological symptoms or signs appeared during this period, cranial ultrasounds and/or MRIs were performed in a timely manner. For children hospitalized owing to other symptoms (e.g., jaundice and infection), cranial ultrasound and/or MRIs were performed as a routine procedure. All the children in the study group met the inclusion criteria described below and their diagnoses were confirmed by MRIs.

The inclusion criteria were: (1) MRI performed during the neonatal period and the diagnosis of a stroke; in this study, hemorrhagic strokes refer to cerebral parenchymal hemorrhages in full-term infants and near-term infants over 35 weeks of gestational age (12), while ischemic strokes include arterial ischemic strokes and neonatal intracranial venous sinus thrombosis (13); (2) the maternal pregnancy examinations were completed in Beijing; (3) the date of birth of the neonate was between March 1, 2019 and February 29, 2020; (4) the place of birth was a unit involved in this study; and (5) the legal guardian signed the informed consent form.

The exclusion criteria were: (1) subdural, epidural, or subarachnoid hemorrhages; (2) germinal matrix or intraventricular hemorrhages in premature infants; and (3) refusal to sign the informed consent form.

### Control Group

The researchers matched the control group to the study group according to gestational age (with a difference in gestational age of 3 days) and date of birth (with a difference of 2 weeks from the study group, at a ratio of 1:4).

The control group also attended follow-ups and met the inclusion criteria below.

The inclusion criteria were: (1) all the maternity examinations during pregnancy were completed in Beijing; (2) the date of birth of the neonate was between March 1, 2019 and February 29, 2020; (3) the place of birth was a unit involved in this study; (4) the imaging examination (cranial ultrasounds and/or cranial MRIs) was normal; and (5) the legal guardian signed the informed consent form.

The exclusion criteria were: (1) brain injury and abnormal brain development caused by other etiologies (e.g., hypoxic-ischemic encephalopathy, bilirubin encephalopathy, hypoglycemic encephalopathy, and brain structural malformations); (2) refusal to undergo brain imaging examination; and (3) refusal to sign the informed consent form.

## Neuroimaging

Owing to economic restrictions and human resource limitations, this study did not perform routine imaging screening on all of the participants in the cohort. Only the participants with neurological symptoms and/or pathological conditions were screened. However, all the patients in the cohort were uniformly followed-up to reduce the missed detection rate.

## Data Collection

Specific data were collected using standardized information forms. First, population information, including the number of live births delivered in each unit per month, was collected. Second, collection included general information, such as gestational age, age, and sex. Third, clinical data, including age at presentation, symptoms, signs, treatment history, and medication, were collected. The fourth set of collected information included the potential risk factors, including maternal factors (age at time of delivery, pregnancy complications, primiparity, and parity), perinatal factors (delivery method, intrauterine distress, and acute intrapartum events, including placental or uterine abruption, postnatal resuscitation, meconium-stained amniotic fluid, and umbilical cord around the neck), and neonatal factors (birth weight and infection status). Fifth, imaging data from all the patients diagnosed with or with difficult to diagnose conditions, comprising a 3.0-T MRI [T1, T2, diffusion-weighted imaging, susceptibility-weighted imaging, and T2 fluid-attenuated inversion recovery (FLAIR) sequence] were collected.

### Data Preservation

The maternity hospitals involved in this study provided the Peking University First Hospital, the leading unit, monthly with data on the delivery volume and detailed information on confirmed and suspected cases. The specific follow-up period was included in the database only after an expert group's diagnosis was verified and sent back to the corresponding unit. The control group was selected according to the final confirmed cases and all the participating units provided the leading unit with detailed information on the control group every month. After the unit obtained information on a case and on a specific follow-up destination, the leading unit contacted the follow-up unit to check the case information and incorporate it into the research process. The researchers archived the data and checked it every 3 months. Any logical errors or missing information were reported to the source unit. All the follow-up information was uploaded to a network database.

## Follow-Up After Birth

The initial plan of this study was to perform outpatient follow-ups at the clinic. However, due to coronavirus disease 2019 (COVID-19) pandemic, some cases required WeChat video calls in addition to the outpatient follow-ups. If the partner hospital did not have the required conditions to perform the follow-up procedures, the leading hospital and/or one of the three neonatal referral hospitals would do so. A chief neonatal physician appointed by each unit and who had more than 10 years of work experience conducted the follow-ups. All the enrolled children were contacted by the pediatrics specialist of the Peking University First Hospital, who then conducted the follow-ups at 1, 3, 6, 9, 12, and 18 months postpartum at the designated chief physician's outpatient clinic.

Follow-ups included clinical manifestation, symptoms, and physical and auxiliary examinations of the children. Neurodevelopmental evaluations were performed using the Ages and Stages Questionnaire (ASQ) for all the enrolled children at 36 months postpartum (14). It covers five areas of child development, each with six items: communication, gross motor, fine motor, problem solving, and personal–social domains. Each item represents a developmental milestone for that specific age group. Items responses are based on a three-item scale that showed the capacity of the child to perform that item, with “yes” being assigned 10 points, “sometimes” being assigned five points, and “not yet” being assigned zero points. The sum of the six items for one area forms the area score. This score then aids with figuring out whether the child is developing normally, at risk of suspected developmental delays (borderline), and a diagnosis of developmental delays in an area. Development delays in communication, gross motor, fine motor, problem solving, and personal–social domains were defined as a score below 13.06, 37.38, 34.32, 25.74, and 27.19. Meanwhile, suspected developmental delays in the above five items were defined as a score below 29, 46, 44, 36, and 38, respectively.

The Bayley Scales of Infant and Toddler Development, third edition (Bayley-III), was used for the diagnostic evaluation, if the ASQ results were abnormal (15). Neurodevelopment delays were defined as the Bayley-III cognitive score of <85 or the Bayley-III motor composite score of <85.

## Quality Control

### Diagnosis

The leading unit provided all the cooperating units with diagnostic training by videos and meetings and explained the standards. The cranial MRIs of all the newborns whose diagnosis was difficult were performed by two radiologists of the leading units. Both have over 5 years of experience in reviewing neonatal neurological diseases. Imaging specialists conducted a blind review. The diagnosis was confirmed or excluded only when the neonatal imaging team (which consists of two neuroimaging specialists with more than 10 years of experience from the imaging department of Peking University) agreed. Upon non-agreement, the team discussed the case further until reaching a consensus.

### Data Collection

Each cooperating unit arranged fixed data collectors. When the project started, all the cooperating units received training on the process to be followed in terms of the case data collection. The monthly delivery volume reported by the 16 hospitals was verified and recorded and the contents of the various cases' data on the strokes reported by all the cooperating units were verified and feedback every 3 months.

#### Follow-Up

The leading hospital designed a standardized follow-up manual and distributed it to all the units expected to conduct them. All the professional physicians involved in the follow-up and who worked in units that had the ability to conduct the ASQ and the Bayley scale were trained on the follow-up procedures before the project began.

#### Project Progress

The project leader of each cooperating unit monitored the progress longitudinally. They communicated with the leading unit through an online meeting every 3 months to clarify the project progress and conducted offline or online meetings with personnel from all the centers every 6 months. The leading unit reported on project progress and supplied feedback on the problems occurring in the research process of the partner hospitals during the meeting that took place every 3 months.

## Statistical Analysis

Numerical data were expressed as frequencies (percentages) and analyzed using the chi-squared tests. Measurement data were expressed as median (minimum-maximum) or mean ± SD and were analyzed by *t*-tests. The logistic regression analysis was applied to the risk factors. Statistical significance was set at *p* < 0.05. The researchers analyzed the statistical data with SPSS software version 26.0.

## Results

### Demographics and Incidence

During the study period, 71,817 live births were delivered by all the cooperating units, including 42,919 live births in the central area and 28,898 live births in the suburbs. The number of live births in the spring, summer, autumn, and winter seasons were 18,802, 18,186, 18,954, and 17,875, respectively.

A total of 27 cases (Table 1) of perinatal strokes were diagnosed in 14 male (52%) and 13 female (48%) neonates. Among the confirmed cases, 15 had hemorrhagic and 12 had ischemic strokes (seven cases of arterial ischemic stroke and five cases of venous sinus thrombosis). Eighteen cases were from the central areas of the city and nine cases were from the suburbs. Spring and summer each saw eight diagnosed cases of perinatal strokes, while autumn saw six diagnosed cases of perinatal strokes and winter saw only five diagnoses cases of perinatal strokes.

**Table 1:** Patients' characteristics.

**Stroke**	**Case**	**Sex**	**Stroke territory**	**Co-morbidities**	**Outcome**
Neonatal arterial ischemic stroke	1	F	Anterior LMCA	Asphyxia	N
	2	M	Posterior LMCA	Dehydration; Cholestasis; IDM	N
	3	M	Anterior LMCA	Dehydration	N
	4	M	Main RMCA	Asphyxia	Epilepsy
	5	F	Posterior LMCA	Sepsis; Twins	N
	6	M	Middle LMCA	Hyperbilirubinemia	N
	7	F	Main LMCA	Neonatal infection	N
Cranial venous sinus thrombosis	8	F	Thalamus	IVH	N
	9	M	Superior sagittal sinus	Bacterial meningitis; Sepsis; Pneumorrhagia	N
	10	M	Bilateral transverse sinus	Sepsis (GBS); ARDS; AKI; Thrombocytopenia	N
	11	M	Bilateral sigmoid sinus	Sepsis; Asphyxia; AKI; Coagulation disorders; LGA	N
	12	M	Bilateral transverse sinus	Neonatal pneumonia (Klebsiella pneumoniae); Subarachnoid hemorrhage; Coagulation disorders	N
Neonatal hemorrhagic stroke	13	F	Cerebellum	IDM; LGA; Subarachnoid hemorrhage; Subdural hemorrhage	N
	14	F	Cerebellum	Hyperbilirubinemia; Craniotabes	N
	15	M	Cerebellum	Neonatal pneumonia	N
	16	M	Cerebellum	Hyperbilirubinemia; Subdural hemorrhage	N
	17	M	Frontal lobe	Dehydration; Hypernatremia; Metabolic acidosis; Subdural hemorrhage; IVH; Subarachnoid hemorrhage	Motor delay
	18	F	Cerebellum; Temporal lobe; Occipital lobe	Asphyxia; Metabolic acidosis; Cephalohematoma; IDM	N
	19	F	Temporal lobe; Cerebellum	Hypernatremia	N
	20	F	Frontal lobe	Subdural hemorrhage; IDM	N
	21	M	Temporal lobe	Asphyxia; Skull fracture; Subdural hemorrhage; Epidural hemorrhage; Subarachnoid hemorrhage; LGA	Motor and communication delay
	22	M	Temporal lobe	IDM	N
	23	F	Parietal lobe	VSD; PDA; IDM; Metabolic acidosis	N
	24	M	Temporal lobe	Neonatal pneumonia	N
	25	M	Frontal lobe; Cerebellum; Basal ganglia	SGA	N
	26	F	Temporal lobe; Occipital lobe	Craniotabes	N
	27	M	Frontal lobe	ARDS; PPHN	N

*LMCA, left middle cerebral artery; RMCA, right middle cerebral artery; F, female; M, male; IDM, infant of diabetic mother; IVH, intraventricular hemorrhage; GBS, group B streptococcus agalactiae; ARDS, acute respiratory distress syndrome; PPHN, persistent pulmonary hypertension of the newborn; AKI, acute kidney injury; LGA, large for gestational age; SGA, small for gestational age; VSD, ventricular septal defect; PDA, patent ductus arteriosus; N, normal*.

Based on this calculation, the incidence of perinatal stroke in Beijing was one in 2,660 live births. In urban areas, this incidence was one in every 2,384 live births and one in 3,210 live births in suburban areas. The incidence rates in spring, summer, autumn, and winter were 1/2,350, 1/2,273, 1/3,159, and 1/3,175, respectively. For perinatal ischemic strokes, the incidence was 1/5,985 and 1/4,788 for hemorrhagic strokes.

### Clinical Features

#### Onset Time of Symptoms

Among neonates with acute symptomatic perinatal strokes (17 neonates), some who had convulsions started to experience the symptoms within 12 h after birth (two neonates). Most symptoms occurred between 12 and 72 h after birth (eight neonates).

There were eight cases of convulsions among neonates with ischemic stroke, with six of them developing their symptoms within 12–72 h after birth. Of these eight patients, six patients had arterial ischemic strokes: five patients involving the left middle cerebral artery and one patient involving the right middle cerebral artery. Additionally, two cases had venous sinus thrombosis involving the bilateral transverse sinuses and sigmoid sinuses, respectively. Of the two cases who developed symptoms within 12 h after birth, one case had an arterial ischemic stroke involving the left middle cerebral artery and the other had a venous sinus thrombosis involving the bilateral sigmoid sinuses.

Among the patients with hemorrhagic stroke, two cases showed convulsions, both of which had symptom onset within 12–72 h after birth. The one case involved the right frontal lobe and the other case involved the left temporal lobe simultaneously.

#### Onset Symptoms

Of the 27 perinatal stroke cases, 17 (62.96%) patients had acute symptomatic strokes. The onset symptoms included convulsions (10, 37.04%), muscular hypertonia (3, 11.1%), frequent apnea (2, 7.40%), crying sharply (1, 3.70%), and decreased responsiveness (1, 3.70%). Routine cranial ultrasound examinations were performed for other children who were diagnosed with other conditions. These included perinatal hyperbilirubinemia, hypoxic asphyxia, and central nervous system infection and the diagnosis was further confirmed by cranial MRIs (Table 2).

**Table 2:** MRI features of neonatal perinatal strokes.

**Stroke territory**	**NIS**	**NHS**
	***n* (%)**	***n* (%)**
BGT	2 (8)	1 (5)
PLIC	1 (4)	-
Central sulcus	1 (4)	-
Superior sagittal sinus	1 (4)	-
Transverse sinus	2 (8)	-
Sigmoid sinus	1 (4)	-
Cerebellum	-	7 (33)
Frontal lobe	4 (15)	4 (19)
Temporal lobe	5 (19)	6 (29)
Parietal lobe	4 (15)	1 (5)
Occipital lobe	5 (19)	2 (10)

*BGT, basal ganglia and thalami; PLIC, posterior limb of the internal capsule; NIS, neonatal ischemic stroke; NHS, neonatal hemorrhagic stroke*.

Among the children with ischemic strokes, 75% (9/12) patients had an acute symptomatic stroke, of which 66.67% (8/12) patients suffered from convulsions. Five patients had a stroke involving the left middle cerebral artery. In one case, the right middle cerebral artery, bilateral transverse sinuses, and bilateral sigmoid sinuses were also involved. Only one (out of 12; 8.33%) patient showed muscular hypertonia and hemorrhage of the left thalamus. The other three cases were diagnosed based on the results of a conventional cranial ultrasound and their strokes involved the left middle cerebral artery, superior sagittal sinus, and bilateral transverse sinuses.

Among children with hemorrhagic strokes, 53.5% (8/15) presented with an acute symptomatic stroke, of which two cases had convulsions. Of these two, one involved the right frontal lobe and the other involved the right temporal lobe. Two patients had hypertonia, with one case involving multiple parts of the temporo-occipital lobe and cerebellum and the other case involving the left ventricle and left frontal lobe (at the same time). Two patients had frequent apnea, with one case involving the left frontal lobe and the other case involving the two hemorrhage sites in multiple parts of the lateral frontal lobe, left basal ganglia, and bilateral cerebellum. In one case, the patient displayed symptoms of crying sharply, vomiting, and the stroke involved the left frontal lobe. Another patient showed irritability and the stroke involved the bilateral cerebellum.

The remaining seven cases of hemorrhagic strokes with no neurological symptoms were diagnosed based on the results of a routine cranial ultrasound. Additionally, they had a history of perinatal hypoxia and asphyxia, were children older than gestational age, younger than gestational age, born from diabetic mothers, and had hyperbilirubinemia. The left cerebellum (*n* = 2), bilateral cerebellar left temporal lobe (*n* = 1), bilateral cerebellum (*n* = 1), left temporal (*n* = 1), left parietal (*n* = 1), and occipital lobes (*n* = 1) were involved in these seven cases.

### Risk Factors

The risk factors in the study and control groups are shown in Table 3. Results showed that the following factors were related to perinatal stroke: the maternal factor of primiparity; the perinatal factors of placental abruption/acute chorioamnionitis, premature rupture of membranes ≥ 18 h, intrauterine distress, and asphyxia/resuscitation; and the neonatal factor of severe infection (i.e., sepsis, bacterial meningitis). Of the 27 neonates in the study group, 23 (85%) had one or more of these factors, the most common was a history of perinatal hypoxia (e.g., having presented intrauterine distress or postnatal asphyxia; 16 cases, 59%). Among the 108 neonates in the control group, 35 (32%) had at least one of these factors (*p* < 0.05).

**Table 3:** Analysis of the risk factors of perinatal strokes by maternal, perinatal, and neonatal factors and groups.

	**Study group**	**Control group**	***X* ^2^**	***P***
	***n* (%)**	***n* (%)**		
**Maternal factors**				
Mother's age ≥ 35 years	4 (15)	33 (31)	2.690	0.077
Diabetes	6 (22)	22 (20)	0.045	0.508
Hypertension	3 (11)	14 (13)	0.067	0.546
Autoimmune disease	1 (4)	7 (6)	0.299	0.697
Multiple pregnancy	1 (4)	4 (4)	0.000	0.739
Primiparity	15 (56)	36 (33)	4.538	0.045
Gestational age <35 weeks	1 (4)	4 (4)	0.000	0.739
**Perinatal factors**				
Cesarean delivery	11 (41)	46 (43)	0.030	0.52
MSAF	6 (22)	16 (15)	0.869	0.254
Umbilical cord around neck	3 (11)	8 (7)	0.396	0.383
PROM ≥ 18 h	6 (22)	4 (4)	10.800	0.01
Acute chorioamnionitis	2 (7)	1 (1)	7.793	0.025
Intrauterine distress	16 (59)	29 (27)	10.208	0.002
Asphyxia / resuscitation	12 (44)	9 (8)	21.443	0.000
**Neonatal factors**				
Male	13 (48)	53 (49)	0.007	0.552
SGA / LGA	4 (15)	19 (18)	0.118	0.493
Severe infection	4 (15)	2 (2)	8.547	0.015

*PROM, premature rupture of membranes; MSAF, meconium-stained amniotic fluid; SGA, small for gestational age; LGA, large for gestational age*.

Based on the results of this univariate logistic regression analyses, all the aforementioned-related factors were subjected to the binary logistic regression analysis. The results demonstrated that primiparity, premature rupture of membranes ≥ 18 h, intrauterine distress, asphyxia/resuscitation, and severe infection were significantly related to the occurrence of perinatal stroke (details in Table 4). Mothers of infants with perinatal strokes were more likely to be primipara [odds ratio (OR) 3.484; 95% CI 1.032–11.759]. In 22% of patients, premature rupture of membranes ≥ 18 h was diagnosed compared with only 4% in the comparison group (OR 11.359; 95% CI 1.522–84.759). Cases were more likely to have intrauterine distress (OR 5.479; 95% CI 1.362–22.041), asphyxia/resuscitation (OR 7.001; 95% CI 1.657–29.589), and suffer from severe infection (OR 12.065; 95% CI 1.123–129.611).

**Table 4:** The logistic regression analysis of risk factors for perinatal strokes.

**Factors**	***P***	**OR**	**95% CI**
Primiparity	0.044	3.484	1.032~11.759
Intrauterine distress	0.017	5.479	1.362~22.041
PROM ≥ 18 h	0.018	11.359	1.522~84.795
Asphyxia / resuscitation	0.008	7.001	1.657~29.589
Severe infection	0.000	12.065	1.123~129.611

*PROM, premature rupture of membranes; OR, odds ratio; CI, confidence interval*.

### Prognosis

All the patients were followed-up regularly until 18 months of age. Twenty-six patients completed the ASQ-3 assessment and one completed the Bayley scale. In the study group, 11.1% (3/27) of the patients had adverse neurodevelopmental outcomes. One patient had an ischemic stroke accompanied by convulsions and seizures within 12 h after birth, which affected the right middle cerebral artery. This patient was treated with oral antiepileptic drugs and had no clinical seizures. However, his electroencephalogram (EEG) was still abnormal at 18 months of age.

The other two patients had hemorrhagic strokes. One patient, whose parents withdraw the treatment shortly after birth, had a right frontal hemorrhage (4.9 × 5.9 cm) and the ASQ scores showed that gross and fine movements were below the threshold. The other patient suffered from a hemorrhage in the right temporal lobe (0.9 × 0.7 cm) and their ASD scores showed that the gross and fine movements and communication dimensions were below the threshold.

The ASQ scores of children in both the groups showed that patients who had convulsions had lower scores than those in the control group for total scores, communication, gross motor, and fine motor dimensions (Table 5) and the difference was statistically significant (*p* < 0.05).

**Table 5:** The Ages and Stages Questionnaire (ASQ) scores according to stroke type and symptoms.

**ASQ dimension**	** $\bar{\text{X}}$ **	**σ**	***T***	***P***
**Total scores**				
Ischemic	249.58	15.87	−0.663	0.514
Hemorrhagic	256.33	32.21		
Convulsion	239.00	9.45	−2.253	**0.033**
Non-convulsion	263.82	6.38		
Symptom onset time <12 h	246.25	31.48	−0.671	0.516
Symptom onset time within 12–72 h	255.00	20.91		
**Communication**				
Ischemic	47.92	6.20	−0.535	0.598
Hemorrhagic	49.67	10.60		
Convulsion	43.50	8.51	−2.305	**0.030**
Non-convulsion	50.88	7.75		
Symptom onset time <12 h	45.83	10.62	−1.144	0.277
Symptom onset time within 12–72 h	51.00	7.41		
**Gross motor**				
Ischemic	51.67	2.46	0.000	1
Hemorrhagic	51.67	7.94		
Convulsion	48.00	7.14	−2.353	**0.036**
Non-convulsion	53.82	4.15		
Symptom onset time <12 h	50.00	7.69	−0.882	0.407
Symptom onset time within 12–72 h	52.00	4.47		
**Fine motor**				
Ischemic	51.67	2.46	0.000	1
Hemorrhagic	51.67	7.94		
Convulsion	45.00	5.27	−3.722	**0.002**
Non-convulsion	52.35	4.37		
Symptom onset time <12 h	47.92	6.89	0.000	1
Symptom onset time within 12–72 h	48.00	4.47		
**Problem solving**				
Ischemic	50.00	6.03	−0.459	0.651
Hemorrhagic	51.00	5.07		
Convulsion	50.50	6.43	−0.808	0.433
Non-convulsion	52.35	4.37		
Symptom onset time <12 h	50.83	6.33	−1.111	0.347
Symptom onset time within 12–72 h	52.00	4.47		
**Personal-social**				
Ischemic	49.58	6.20	−1.556	0.135
Hemorrhagic	53.00	4.92		
Convulsion	49.50	5.99	−1.372	0.188
Non-convulsion	52.64	5.34		
Symptom onset time <12 h	51.67	6.15	−0.107	0.917
Symptom onset time within 12–72 h	52.00	5.70		

*Bold values represents P < 0.05*.

## Discussion

This multicenter prospective study described the incidence and clinical characteristics of perinatal strokes in Beijing, with the aim to provide a basis for diagnosis and prognosis improvement. The incidence was 1 in 2,660 live births, which is consistent with earlier reports (1/1,600–1/3,000) (2). There was no statistically significant difference in incidence between regions, nor among seasons, which might be related to the limited number of cases in this study.

Previous studies have reported that arterial ischemic strokes are the most common type of stroke in neonates, but the evidence in this study showed that the incidence for hemorrhagic strokes was higher (2). This may be due to the routine neuroimaging procedures for patients with no neurological symptoms in this study, as was the case for approximately half of the neonatal hemorrhagic stroke cases in this study. Instead, these patients, who were diagnosed with perinatal strokes without neurological symptoms, had been hospitalized for neonatal hyperbilirubinemia and perinatal hypoxia and asphyxia. Through the routine use of bedside cranial ultrasounds, the strokes were found coincidently; this urged the unit team to run a brain MRI, which then confirmed the diagnosis. In addition, three asymptomatic patients (25% of patients with ischemic strokes in the study sample) were diagnosed through screening. This indicates that neonates with risk factors such as severe infection or perinatal hypoxia should be routinely examined by a cranial ultrasound, as this may ensure that they are diagnosed early and receive timely interventions. These results also show that there may be cases in which hemorrhagic strokes were missed in neonates because of the lack of neurological manifestations. Therefore, subsequent studies should further expand the scope of screening to obtain more accurate data.

This study showed that the most common location involved in the stroke was the left middle cerebral artery, consistent with the literature results (16). This may be related to the characteristics of fetal circulation. Specifically, at first, either a placental thrombosis can enter the umbilical vein or an embolus can form locally in the heart. Then, either one can pass through the foramen ovale and eventually enter the left cerebral artery or directly enter the left cerebral artery through the foramen ovale, as described by prior research (17). Despite these suspicions, research shows that the etiology of neonatal hemorrhagic strokes is unclear and that the most common lesion of such strokes is in the temporal lobe (18). In this study, 40% of patients with hemorrhagic strokes had temporal lobe hemorrhages. At the same time, a relatively high proportion of patients (46.7%) had cerebellar hemorrhages and most patients were asymptomatic and diagnosed by routine cranial ultrasound screenings. Although small-volume cerebellar hemorrhages have a relatively good prognosis, those that experienced them may develop dysplasia, including motor and language impairment at about 2 years of age (19). This fact once again suggests the need for actively screening high-risk patients while paying attention to the presence of cerebellar hemorrhages, which can be imaged through the additional mastoid fontanelle windows of ultrasounds.

It has been reported in the literature that primiparity, intrauterine distress, asphyxia, and postnatal resuscitation are closely related to perinatal strokes (18, 20–25). Intrauterine distress and asphyxia often lead to the hypoxia-ischemia procedure and temporary hypoxia can lead to vasomotor dysfunction, damage to the vascular endothelium, and hypercoagulability. These complications are the cause of hemorrhages or ischemia in the arteries or veins after hypoxia (20–23). This study showed that 59% of patients in the study group had a history of intrauterine distress, asphyxia, or postnatal resuscitation and this rate was significantly higher than that in the control group (27%). Primiparity may contribute to prolonged labor, which increases the risk of perinatal hypoxia and asphyxia (20). Furthermore, infection is a common risk factor for perinatal stroke (25, 26). In this study, two patients in the study group had acute chorioamnionitis owing to placental pathology. Additionally, the proportion of patients with severe infections was significantly higher in the study group than in the control group. Acute chorioamnionitis is likely to release cytokines, causing diffuse arterial injury, thrombosis, and intracranial arteritis in the fetus (23, 27, 28). Severe infections can also induce vascular endothelial injury and hypercoagulability (1). Further, the results showed that the risk of a perinatal stroke was 12.065 times higher in the case group than in the control group. Regarding other complications, 22% of patients in the study group had a history of premature rupture of membranes and this proportion was significantly higher than that in the control group (4%). Among children with premature rupture of membranes in both the groups, the risk of perinatal strokes was 11.359 times higher in the case group than that in the control group. Indeed, earlier investigations have found that inflammatory markers in amniotic fluid are related to perinatal strokes (29). Thus, this study speculates that the premature rupture of membranes causes inflammation and results in a perinatal stroke. However, this pathophysiology requires further laboratory tests and clinical evidence.

In general, patients with perinatal strokes have a poor prognosis, as 9–40% of them show adverse outcomes (6–8). Noticeably, some patients with stroke show developmental abnormalities after 2 years of age (2, 16, 30, 31). In this study, 11% of the neonates showed adverse neurodevelopmental outcomes, denoting that the rate was generally lower than that reported in the literature. This may be related to the early detection and active follow-up enabled by the procedures taken in this study and that were proposed despite the children going through an asymptomatic period. It was early detection and active follow-ups that facilitated the early detection of neurological disabilities and timely rehabilitation. However, this study had a follow-up period of 18 months after birth, thus could not assess these abnormalities. Longer follow-up periods are required in future studies.

## Study Limitations

In this study, not all the children had a routine neuroimaging follow-up and, therefore, the actual incidence of perinatal strokes may have been higher. Besides, the relatively small number of children with perinatal strokes in this study may limit the assessment of risk factors and outcomes. Furthermore, the relatively short duration of the follow-up limits long-term outcome assessments. In addition, the researchers compared perinatal strokes in two categories ischemic stroke and hemorrhagic stroke, which make comparisons with other studies difficult.

## Conclusion

In this study, the incidence of perinatal strokes in Beijing was found to be one in 2,660 live births and the first symptom in most newborns within 12–72 h after birth was convulsions. The results highlight how cranial neuroimaging may be used to detect asymptomatic strokes in neonates with associated risk factors, especially hemorrhagic strokes. Further, primiparity, intrauterine distress, asphyxia, resuscitation, premature rupture of membranes over 18 h, and severe infections are all high-risk factors for perinatal strokes and the most common risk factor is perinatal hypoxia. Patients diagnosed with a perinatal stroke are at high risk for developing neurological sequelae. Therefore, early screening, treatment after diagnosis, regular follow-ups, and rehabilitation are important to improve their prognosis.

## Data Availability Statement

The raw data supporting the conclusions of this article will be made available by the authors, without undue reservation.

## Ethics Statement

The studies involving human participants were reviewed and approved by Peking University First Hospital Ethics Committee. Written informed consent to participate in this study was provided by the participants' legal guardian/next of kin. Written informed consent was obtained from the individual(s) and minor(s)' legal guardian/next of kin, for the publication of any potentially identifiable images or data included in this article.

## Author Contributions

QX, ZhY, and YX collected and analyzed the data, wrote the manuscript, and conducted statistical analysis. YZ was responsible for the interpretation of the MRI results. ZiY, MH, YD, WK, LK, SY, YM, ZF, LZhe, YLe, PW, JD, LY, JJ, HC, SZ, JY, PZ, RW, HG, XinlL, HT, YLi, ZZ, LZha, JY, ZL, CY, QW, JW, LS, HZ, JL, WQ, XW, GL, and XY provided information on the number of births and/or data of patients in their respective hospitals. XinxL, XZ, XX, YG, HL, and LZha participated in discussions surrounding the project design and implementation and provided suggestions. LL, ZT, and XH designed the research and supervised the implementation, guided the data analysis, and revised the article. All the authors have contributed to the manuscript and approved the submitted version of the manuscript.

## Funding

The Beijing Municipal Science and Technology Commission (No. Z191100006619049 to XH) and the Beijing Municipal Science and Technology Commission (No. Z211100002921050 to LL) supported this study.

## Conflict of Interest

The authors declare that the research was conducted in the absence of any commercial or financial relationships that could be construed as a potential conflict of interest. The handling editor declared a shared affiliation with several of the authors WQ and XW at time of review.

## Publisher's Note

All claims expressed in this article are solely those of the authors and do not necessarily represent those of their affiliated organizations, or those of the publisher, the editors and the reviewers. Any product that may be evaluated in this article, or claim that may be made by its manufacturer, is not guaranteed or endorsed by the publisher.
